# Immunogenicity of inactivated COVID-19 vaccine in patients with autoimmune inflammatory rheumatic diseases

**DOI:** 10.1038/s41598-022-22839-0

**Published:** 2022-10-26

**Authors:** Yi-Qing Zheng, He-Jun Li, Ling Chen, Shun-Ping Lin

**Affiliations:** Department of Rheumatology, Fujian Medical University Union Hospital, Fuzhou, China

**Keywords:** Inactivated vaccines, Rheumatology

## Abstract

Progress has been made in COVID-19 vaccine development, with encouraging safety and efficacy data. The purpose of this study was to investigate the immunogenicity of inactivated COVID-19 vaccine in patients with autoimmune inflammatory rheumatic diseases (AIIRD). Patients with AIIRD (n = 101) were included in this study. All patients received 2 doses of inactivated COVID-19 vaccine. Serum anti-S1/RBD protein IgG was detected 2–16 weeks after the second vaccination. Seropositivity was defined as IgG ≥ 1.00 bound antibody unit S/CO. Immunogenicity of inactivated COVID-19 vaccine was assessed by seropositivity rate and the levels of serum IgG antibody against anti-S1/RBD protein, compared with the general population (n = 46). There was no difference by statistical significance in the seropositivity rate between patients with AIIRD (82.2%) and SLE (86.1%) and the control group (93.5%), *p* > 0.05. The level of anti-S1/RBD protein IgG antibodies in patients with AIIRD (median [IQR], 8.8 [2.2–17.3]) and SLE (median [IQR], 9.6 [2.4–20.4]) was comparable to that in the control group (median [IQR], 7.2 [3.1–14.2]), *p* > 0.05. Patients treated with glucocorticoids(GCs) (median dose, [IQR]: 2.5 mg/day [IQR 2.5–5.0]) or hydroxychloroquine(HCQ) or GCs + HCQ without other immunomodulatory medications, had an appropriate immunogenic response(88.1%) with high levels of anti-S1/RBD protein IgG(median [IQR], 12.1 [6.5–20.4]). Neither of patients treated with rituximab had positive serum antibodies, which was statistically significant, compared with the control group (*p* < 0.01). Compared with the control group, methotrexate(MTX) and iguratimod(IGU) was significantly reduced the level of anti-S1/RBD protein IgG antibodies. Inactivated COVID-19 vaccine had appropriate immunogenicity in patients with AIIRD. Immunogenicity of inactivated COVID-19 vaccine was severely impaired by rituximab, and also suppressed by MTX and IGU, while low doses of GC and HCQ had negligible effect.

## Introduction

Since the outbreak of COVID-19, several vaccines against COVID-19 have been approved for emergency use or conditional sale in many countries,and showed high efficacy against severe disease. The inactivated COVID-19 vaccine showed high safety and immunogenicity in preliminary clinical trials^1^ and the mid-term analysis of randomized, double-blind, placebo-controlled phase III clinical trials ^2^. The serum positive conversion rates in the two vaccine groups were 99.3% and 100.0% respectively^3^. The inactivated COVID-19 vaccine developed in China has been used in the wider population since December 2020, and has shown good safety and efficacy in the general population. Patients with autoimmune inflammatory rheumatic diseases (AIIRD) have been prioritised for urgent vaccination to mitigate COVID-19 risk, consistent with the American College of Rheumatology (ACR) Guidance^4^. The latest study shows that the inactivated COVID-19 vaccine is well tolerated in the AIIRD population^5^. However, data on the efficacy of the inactivated COVID-19 vaccine in patients with AIIRD are still scarce. At the same time, Glucocorticoids (GCs), Hydroxychloroquine (HCQ) and other immunosuppressants are widely used in patients with AIIRD. According to current studies, these drugs have certain effects on the immunogenicity of vaccines. Therefore, we conducted an observational study to evaluate the immunogenicity of inactivated COVID-19 vaccine in patients with AIIRD.

## Patients and methods

### Study cohort and patients

This was a retrospective case–control study on the Chinese Han population. Consecutive adult patients (aged ≥ 18 years) who visited the rheumatology outpatients departments of Fujian Medical University Union Hospital during July 2021 to October 2021 were recruited into the study according to the following inclusion criteria: (1) definitively diagnosed with AIIRD; (2) 2–16 weeks after the second inactivated Covid-19 vaccine dose. The control group included a sample of the general population, consisting mainly of healthcare personnel. Exclusion criteria for all groups were pregnancy, lack of informed consent, and previous COVID-19 infection and for controls—history of AIIRD and immunomodulatory medications.

## Methods

The inactivated vaccine was Covilo (0.5 ml/syringe,contain inactivated novel Coronavirus protein 4ug,Beijing Institute of Biological Products Co., LTD, People's Republic of China) or CoronaVac (0.5 ml/syringe,single dose of 0.5 ml contains 1200SU antigen of SARS-CoV2,Sinovac Life Sciences Co., LTD, People's Republic of China).

Demographic (age, sex, comorbidity), disease-specific(type of AIIRD and treatment), disease duration, and vaccination (date, type of vaccine) data were recorded. The interval between the two vaccines and between vaccination and detection of antibody were calculated. At the same time, we recorded the use of immunomodulatory medications in AIIRD patients from 4 weeks before vaccination until antibody testing,including GCs, HCQ, Thalidomide (Thd), cyclosporinA(CSA), Tacrolimus (Tac), Mycophenolate Mofetil (MMF), Methotrexate (MTX), Iguratimod and leflunomide(LEF). In addition, we recorded the use of rituximab in AIIRD patients prior to vaccination. The serum IgG antibody levels against COVID-19 S1/RBD protein were detected by magnetic particle chemiluminescence immunoassay using COVID-19 antibody detection kit (Autobio Diagnostics Co., LTD.).Seropositivity was defined as IgG ≥ 1.00 bound antibody unit S/CO,indicating immunogenicity. The influence of drugs on the immunogenicity of AIIRD patients was assessed by comparing seropositivity rate and the serum IgG antibody levels against COVID-19 S1/RBD protein between groups.The study was conducted at the Rheumatology Department of Fujian Medical University Union Hospital in China and has full ethical approval from the ethics board of Fujian Medical University Union Hospital. We confirm that all methods were performed in accordance with the relevant guidelines and regulations.Informed consent was obtained from all final participants.

### Statistical analysis

Dichotomous variables were expressed as absolute frequencies (percentages) and compared using Chi-square test or Fisher’s exact test. Continuous data were expressed as mean ± SD (standard deviation), or medians [interquartile range (IQR)], and statistical comparison between the groups was calculated with the Mann–Whitney nonparametric U test. *p* values less than 0.05 were considered indicative of statistical significance. All statistical calculations were performed using SPSS statistical software (SPSS Version 21.0).

### Ethics approval and consent to participate

The study was approved by the Ethical Committee of the “Fujian Medical University Union Hospital” in compliance with the ethical principles.

## Results

### Study population

A total of 101 patients with AIIRD, including 79 systemic lupus erythematosus (SLE), 11 rheumatoid arthritis(RA), 4 undifferentiated connective tissue disease(UCTD), 3 primary Sjogren's syndrome(pSS), 2 ankylosing spondylitis(AS) and 2 antineutrophil cytoplasmic antibody (ANCA)-associated vasculitis (AAV), and 46 control group vaccinated with the two-dose inactivated COVID-19 vaccine were enrolled for analysis. There were 91 (90.1%) and 36 (78.3%) females in AIIRD and control group, with a mean age of 37.4 ± 10.8 and 35.0 ± 10.7 years, respectively. The median interval between vaccination and detection of antibody was 37.0 and 35.0 days, respectively. There was no difference by statistical significance in these datas between the two groups(*p* > 0.05). SLE was the most common disease (n = 79) (Table 1).

**Table 1:** Immunogenicity of the inactivated COVID-19 vaccine in patient with AIIRD and controls.

	Age, year, median [IQR]	Gender, F (%)	Disease duration, months, median [IQR]	The interval between the two vaccines, days, median [IQR]	The interval between vaccination and detection of antibody, days, median [IQR]	Seropositivity rate, n (%)	The level of serum anti-S1/RBD protein IgG, S/CO, median [IQR]
Controls group, n = 46	35.0 ± 10.7	36 (78.3%)	N/A	29.0 [23.8–37.8]	35.0 [28.0–69.5]	43 (93.5)	7.2 [3.1–14.2]
AIIRD, n = 101	37.4 ± 10.8	91 (90.1%)	42.0 [18.0–96.0]	27.0 [23.0–32.0]	37.0 [22.0–61.0]	83 (82.2)	8.8 [2.2–17.3]
SLE, n = 79	36.5 ± 9.6	75 (94.9%)	48.0 [19.0–120.0]	26.0 [23.0–31.0]	31.0 [21.0–51.0]	68 (86.1)	9.6 [2.4–20.4]

No difference by statistical significance in these datas between groups (p > 0.05).

*AIIRD* autoimmune inflammatory rheumatic diseases, *SLE *systemic lupus erythematosus, *N/A *not applicable.

### Immunogenicity of the inactivated COVID-19 vaccine

**Figure 1:**
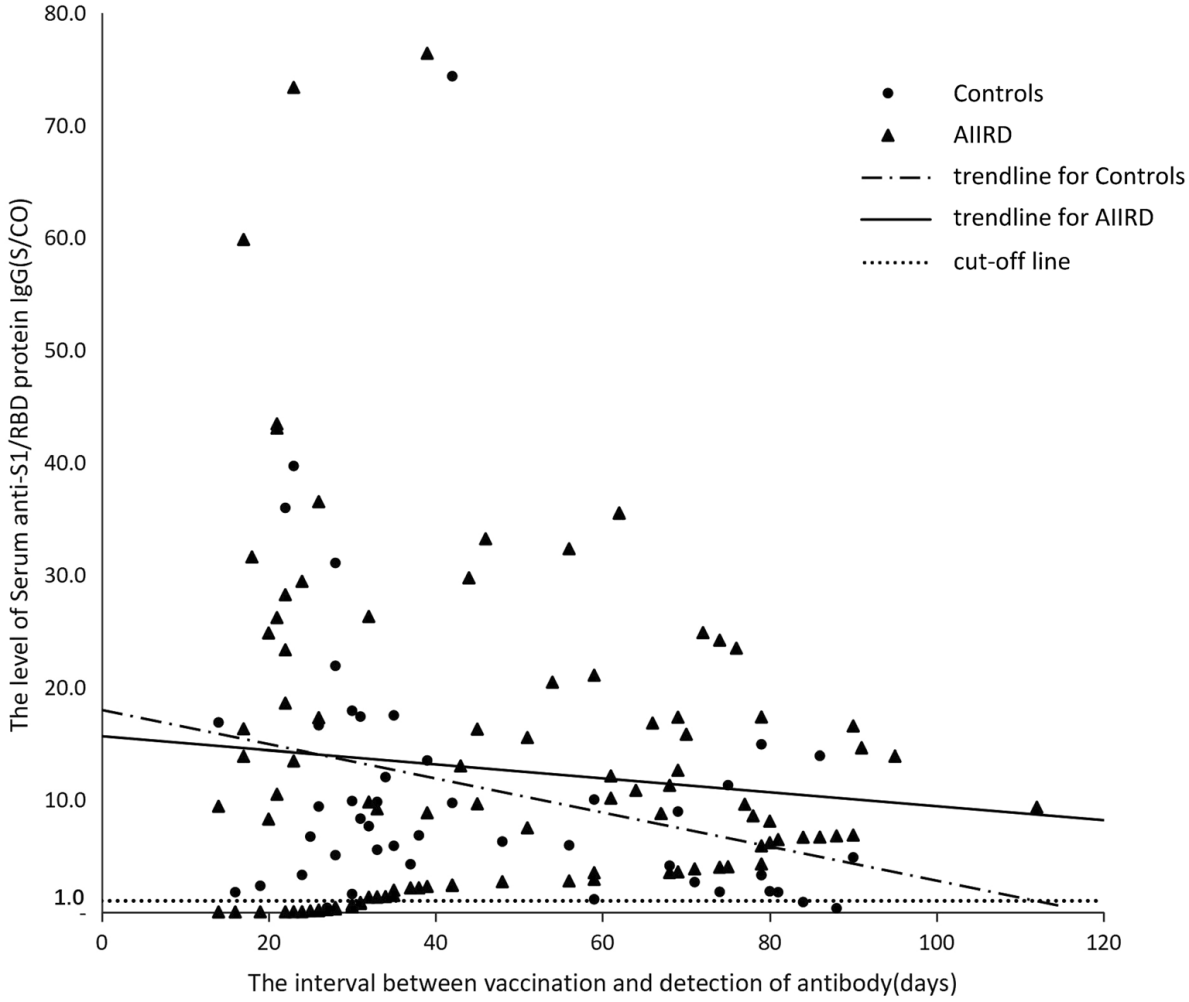
The relationship between the interval between vaccination and detection of antibody and serum IgG levels.

There was no difference by statistical significance in the seropositivity rate between patients with AIIRD (82.2%) and SLE (86.1%) and the control group (93.5%), *p* > 0.05. The level of anti-S1/RBD protein IgG antibodies in patients with AIIRD (median [IQR], 8.8 [2.2–17.3] S/CO) and SLE (median [IQR], 9.6[2.4–20.4] S/CO) was comparable to that in the control group (median [IQR], 7.2 [3.1–14.2] S/CO), *p* > 0.05 (Table 1).The scatter plots of antibody levels in the control group and patients with AIIRD according to the antibody detection time after the second dose of vaccine are shown in Fig. 1.

### Effect of immunomodulatory medications on the immunogenicity of the inactivated COVID-19 vaccine

Glucocorticoids (GCs), and hydroxychloroquine (HCQ) were used in 77.2% (78 out of 101) and 75.2% (76 out of 101) of patients with AIIRD, respectively. GCs or HCQ or GCs plus HCQ without other immunomodulatory medications was used in 58.4% (59 out of 101) of patients with AIIRD. The median dose of AIIRD was 2.5 mg/day (IQR 2.5–5.0) for prednisone and 200.0 mg/day for HCQ (IQR 200.0–200.0) (Table 2). Patients treated with GCs or HCQ or GCs plus HCQ without other immunomodulatory medications, had an appropriate immunogenic response (88.1%) with high levels of anti-S1/RBD protein IgG antibodies (median [IQR], 12.1 [6.5–20.4] S/CO). Both patients treated with rituximab (last dose before vaccination: 100 mg and 400 mg, respectively) had negative serum antibodies, which was statistically significant, compared with the control group (*p* < 0.01). The interval between the last dose of rituximab and vaccination in the two patients was 56 and 63 days respectively. Compared with the control group, methotrexate(MTX) and Iguratimod was significantly reduced the level of anti-S1/RBD protein IgG antibodies (Table 3). Tables 2 and 3 provides additional details.

**Table 2:** Comparison between patients with positive and negative antibody in AIIRD.

	AIIRD patients, n = 101	Positive (n = 83)	Negative (n = 18)	*p* value
Gender, F (%)	91 (90.1)	74 (89.2)	17 (94.4)	0.686
Age, year (mean ± SD)	37.4 ± 10.8	37.0 ± 10.7	39.1 ± 11.2	0.516
Disease duration, months (median, IQR)	42.0 (18.0–96.0)	43.0 (16.0–96.0)	36.0 (22.8–93.0)	0.831
The interval between the vaccine and detection of antibody, day (median, IQR)	37.0 (22.0–61.0)	31.0 (21.0–53.0)	61.0 (32.8–71.0)	0.013
**Immunosuppressive treatments**				
GCs, n (%)	78 (77.2)	64 (77.1)	14 (77.8)	0.105
GCs, mg (median, IQR)	2.5 (2.5–5.0)	2.5 (1.3–5.0)	2.5 (1.9–5.0)	0.647
HCQ, n (%)	76 (75.2)	66 (79.5)	10 (55.6)	0.066
HCQ, mg (median, IQR)	200.0 (200.0–200.0)	200.0 (200.0–200.0)	200.0 (200.0–200.0)	0.758
GCs + HCQ, n (%)	61 (60.4)	54 (65.1)	7 (38.9)	0.510
Thd, n (%)	61 (60.4)	10 (12.0)	1 (5.6)	0.684
Anti-CD20, n (%)	2 (2.0)	0 (0)	2 (11.1)	0.030
CNI, n (%)	9 (8.9)	7 (8.4)	2 (11.8)	0.660
MTX, n (%)	4 (4.0)	2 (2.4)	2 (11.8)	0.145
MMF, n (%)	5 (5.0)	4 (4.8)	1 (5.6)	1.000
Iguratimod, n (%)	5(5.0)	3 (3.6)	2 (11.1)	0.216
LEF, n (%)	2 (2.0)	2 (2.4)	0 (0.0)	1.000

*GCs* glucocorticoids, *HCQ* hydroxychloroquine, *Thd *thalidomide, *CNI *calcineurin inhibitors, *MMF* mycophenolate mofetil, *MTX* methotrexate, *LEF* leflunomide.

**Table 3:** Immunogenicity of the inactivated COVID-19 vaccine according to the use of immunomodulatory medications in comparison with controls.

Immunomodulatory medications, n (%)	Seropositivity rate, n (%)	The level of Serum anti-S1/RBD protein IgG, S/CO, mean ± SD
GCs,78 (77.2)	64 (82.1)	9.3 [2.6–17.3]
HCQ,76 (75.2)	66 (86.8)	9.7 [2.7–20.9]
GCs or HCQ or GCs + HCQ without other immunomodulatory medications, 59 (58.4)	52 (88.1)	12.1 [6.5–20.4]
Thd, 11 (10.9)	10 (90.9)	4.0 [2.2–20.4]
Rituximab, 2 (2.0)	0 (0.0)**	0.02 ± 0.01**
MMF, 5 (5.0)	4 (80.0)	2.2 [0.7–19.5]
CsA, 7 (6.9)	6 (85.7)	4.3 [1.3–23.5]
Tac, 2 (2.0)	1 (50.0)	5.8 ± 7.2
MTX, 4 (4.0)	2 (50.0)	1.7 [0.4–3.7]*
LEF, 2 (2.0)	2 (100.0)	5.7 ± 2.5
Iguratimod, 5 (5.0)	3 (60.0)	1.4 [0.1–5.2]*
No treatment, 5 (5.0)	5 (100.0)	6.2 [4.1–40.8]

*GCs* glucocorticoids, *HCQ *hydroxychloroquine, *Thd *thalidomide, *MMF* mycophenolate mofetil, *CSA *cyclosporin A, *Tac *tacrolimus, *MTX *methotrexate, *LEF* leflunomide.

*p < 0.05.

**p < 0.01.

Two of the 17 antibody negative patients with AIIRD were treated with rituximab, which was statistically significant, compared with the antibody positive patients (no rituximab) (*p* < 0.05) (Table 2).

## Discussions

Prospective studies conducted during the COVID-19 pandemic suggest that COVID-19 vaccine is immunogenic in the majority of AIIRD patients,although somewhat attenuated compared to the general population^6,7^. In our study, 82.2% of AIIRD and 86.1% of SLE patients vaccinated with inactivated COVID-19 vaccine had positive serum antibodies and high levels of the anti-S1/RBD protein IgG antibodies.There was no difference by statistical significance, compared with the control group. The sample size may not be large enough to be significant, but it also reflects only minor differences (if any) between AIIRD patients and controls, suggesting that inactivated COVID-19 vaccine is effective and feasible in patients with stable AIIRD. In a phase 4 trial of CoronaVac inactivated vaccine in autoimmune patients, seropositive rates were 70.4% (vs. 95.5% in controls, *p* < 0.001), which also indicated reduced but acceptable short-term immunogenicity^8^. Compared with this study, the seropositivity rate in AIIRD patients in our study seems to be slightly higher than that in this study. This may be due to the lower dose of GCs [median, (IQR), 2.5 mg (2.5–5.0) vs 5 mg (5–10)], less use of MTX (4% vs 25.5%) and MMF (5% vs 12.8%) in our enrolled patients.

GCs and HCQ are important drugs in the treatment of patients with AIIRD. Study has shown that a dose of GCs ≥ 10 mg/day reduces the humoral response of pneumococcal vaccine in patients with various inflammatory diseases ^9^. In an analysis of 20 SLE patients vaccinated with 13-Valent Pneumococcal Conjugate vaccine treated with hydroxychloroquine (HCQ) showed that hydroxychloroquine (HCQ) did not impair immunogenicity of conjugate pneumococcal vaccine^10^. In our study, patients with AIIRD were treated with GCs doses of less than 10 mg/day. There were a high seropositivity rate and level of anti-S1/RBD protein IgG in patients with AIIRD treated by GCs or HCQ without other immunomodulator, which indicated that low doses of GCs and HCQ had negligible effect on immunogenicity.

In Furer et al.’s study^7^,immunogenicity was severely impaired by rituximab. In our study, there were statistical differences in rituximab use between the antibody negative and positive patients with AIIRD, and neither of patients treated by rituximab had positve anti-S1/RBD protein IgG antibodies in serum. Our findings are in line with the previously published data regarding the negative impact of anti-CD20 therapy on the humoral response to various vaccines^7,11,12^. The interval between rituximab treatment and vaccination plays a critical role in the response to the vaccine. In a retrospective analysis reported by Spiera et al., of 30 patients with rheumatic disease treated with rituximab, only 10 (33.3%) were serologically positive. The seropositivity rate was only 10% among those vaccinated more than 6 months after rituximab treatment, and among those vaccinated 1 year after RTX treatment, the seropositivity rate increased to about 90%. In seropositive patients, the median time from the last dose of rituximab to a second dose of COVID-19 vaccine was 704.5 (IQR 540–1035) days^13^. Among the 74 rituximab-treated patients studied by Mrak et al., the mean time between the last rituximab treatment and the first COVID-19 vaccination was 6.9 ±6.0 months, only 29 (39%) showed seroconversion^14^.

According to current vaccination recommendations^15^, the time between vaccination and the last dose of rituximab should be as long as possible, with enough time to allow the body to develop an immune response to the vaccine. However, since protection against SARS-COV-2 relies on humoral and T-cell-mediated immunity, patients with inadequate humoral response may still be protected by the latter^11,12,14,16–18^.

According to many studies^19–21^, MTX can reduce the immunogenicity and antibody level of vaccine, which is consistent with our results.But in a non-systematic review^22^, it was shown that while MTX reduces the immunogenicity of influenza vaccines and overall antibody titers are low, the titers of vaccines given with MTX in most patients are generally sufficient to protect against influenza infection. Therefore, there is debate about whether and how long MTX should be stopped before and after vaccination. The effect of Iguratimod(IGU) on the immunogenicity of inactivated COVID-19 vaccines is also noteworthy. In our study, the levels of the anti-S1/RBD protein IgG antibodies in five patients who were treated with IGU were significantly lower than that in the control group. Studies have shown that IGU inhibits immunoglobulin (Ig) production in mouse models and in rheumatoid arthritis because it acts directly on B lymphocytes without affecting B cell proliferation^23^. In a prospective controlled study of 50 patients with sjogren's syndrome, serum IgG was significantly reduced in patients treated with IGU^24^. Therefore, IGU may inhibit the immunogenicity of vaccine, which is worthy of further study. Of course, the significance of this study is affected by the small sample size.

In the study conducted by Furer et al.^7^, the levels of the IgG antibody against COVID-19 were significantly lower in patients with AIIRD than the control group. It was considered to be related to the difference in the treatment of the enrolled patients, especially the use of rituximab. Similarly, several studies have shown that rituximab and MTX^6,8^, and even MMF^8,25^ impair immunogenicity to COVID-19 vaccines in the autoimmune systemic diseases. In the Phase 4 Trial of CoronaVac inactivated vaccine in the autoimmune patients, GCs showed to impair the immunogenicity. However, subgroup analyses showed that this impairment occurred only in patients with GCs doses of 20 mg or more per day^8^. Therefore, the main factor affecting the immunogenicity of COVID-19 vaccine in AIIRD may be the immunomodulatory drugs used, rather than the disease itself.

Regarding the use of inactivated COVID-19 vaccines in patients with AIIRD, the study by Fan et al. confirmed the safety profiles, and for the first time demonstrated the disease flare after vaccination^5^, while our study confirmed its immunogenicity. These results provide relatively complete data for patients with AIIRD in the use of inactivated COVID-19 vaccines.

Several limitations should be noted in the interpretation of our results.Firstly, although there were not statistically significant in the between-group comparison,the duration between receiving the second dose till serum analysis, as well as the interval between first and second dose,varied among subjects, due to the absence of strict restrictions, which may have affected the comparison of results. Secondly, controls were mainly health care personnel, which may bias the data slightly.

## Conclusions

The study suggests that considerable immunogenicity was induced by inactivated COVID-19 vaccine in patients with AIIRD, which was affected mainly by the immunomodulatory drugs used in AIIRD. Immunogenicity of inactivated COVID-19 vaccine was severely impaired by rituximab, and also suppressed by MTX and IGU, while low doses of GCs and HCQ had negligible effect.

## Data Availability

The datasets used during the current study are available from the corresponding author on reasonable request.
